# Paresi’s Hæmostatic Collodion

**Published:** 1881-11

**Authors:** 


### PARESI'S HÆMOSTATIC COLLODION.

℞ Officinal collodion . . . ʒ xxv.

Carbolic acid (pure.) . . . ʒ ijss.

Tannic acid . . . . . . ʒ j¼.

Benzoic acid . . . . . . ʒ ¾.

Mix. Second art.

